# Effects of the El Niño-Southern Oscillation on dengue epidemics in Thailand, 1996-2005

**DOI:** 10.1186/1471-2458-9-422

**Published:** 2009-11-20

**Authors:** Mathuros Tipayamongkholgul, Chi-Tai Fang, Suratsawadee Klinchan, Chung-Ming Liu, Chwan-Chuen King

**Affiliations:** 1 Graduate Institute of Epidemiology, College of Public Health, National Taiwan University, Taipei, Taiwan; 2 Division of Infectious Diseases, Department of Internal Medicine, National Taiwan University Hospital, Taipei, Taiwan; 3 Department of Disease Control, Ministry of Public Health, Bangkok, Thailand; 4 Global Change Research Center, National Taiwan University, Taipei, Taiwan

## Abstract

**Background:**

Despite intensive vector control efforts, dengue epidemics continue to occur throughout Southeast Asia in multi-annual cycles. Weather is considered an important factor in these cycles, but the extent to which the El Niño-Southern Oscillation (ENSO) is a driving force behind dengue epidemics remains unclear.

**Methods:**

We examined the temporal relationship between El Niño and the occurrence of dengue epidemics, and constructed Poisson autoregressive models for incidences of dengue cases. Global ENSO records, dengue surveillance data, and local meteorological data in two geographically diverse regions in Thailand (the tropical southern coastal region and the northern inland mountainous region) were analyzed.

**Results:**

The strength of El Niño was consistently a predictor for the occurrence of dengue epidemics throughout time lags from 1 to 11 months in the two selected regions of Thailand. Up to 22% (in 8 northern inland mountainous provinces) and 15% (in 5 southern tropical coastal provinces) of the variation in the monthly incidence of dengue cases were attributable to global ENSO cycles. Province-level predictive models were fitted using 1996-2004 data and validated with out-of-fit data from 2005. The multivariate ENSO index was an independent predictor in 10 of the 13 studied provinces.

**Conclusion:**

El Niño is one of the important driving forces for dengue epidemics across the geographically diverse regions of Thailand; however, spatial heterogeneity in the effect exists. The effects of El Niño should be taken into account in future epidemic forecasting for public health preparedness.

## Background

Dengue fever and dengue hemorrhagic fever is a mosquito-borne disease endemic to Southeast Asia [1-3]. Despite intensive vector control efforts, large periodic dengue epidemics have continued to occur throughout the region in multi-annual cycles [3,4]. The El Niño-Southern Oscillation (ENSO), an ocean-atmosphere phenomenon of the Pacific Ocean with a semi-periodic multi-annual cycle [5-7], has been hypothesized to be a driving force behind the dengue epidemics in regions at risk through its profound influence on the local climate [8,9]. El Niño begins with a rise in surface seawater temperature over the tropical eastern Pacific Ocean that later extends as far as the western Pacific and has a warming effect on surface air [5-7]. Higher air temperature can facilitate the transmission of dengue by increasing the replication rate of the dengue virus in *Aedes aegypti *[10] and the blood-feeding behavior of the mosquito vector [11]. Studies in the Pacific Islands, French Guiana, and Indonesia showed that El Niño was associated with an increase in annual numbers of reported dengue cases [12-14]. A nonstationary association between El Niño dynamics and monthly dengue hemorrhagic fever incidence was also shown in the Bangkok area of Thailand from 1986 to 1992 [8]. From 1995 to 2003, the ENSO cycle was found to predict the subsequent weekly incidence of dengue cases in two municipalities in Mexico with 16-week and 20-week lags, respectively [9].

The temporal sequence between El Niño and the occurrence of dengue epidemics -- an important criterion for causal inference -- has not yet been thoroughly investigated (but see [15]). Alternative explanations for the multi-annual cycles of dengue epidemics have been proposed, such as partial cross-immunity among the four serotypes of dengue virus [16-19]. Moreover, sociological factors such as population structure, unplanned urbanization, international transportation of infected people and mosquitoes may also affect dengue transmission [20-22]. The effect of El Niño on dengue epidemics thus remains controversial [23]. We hypothesized that if the El Niño-Southern Oscillation is a driving force behind dengue epidemics, then ENSO indicators should consistently be predictors for the occurrences of dengue epidemics throughout the biologically plausible range of time lags across geographically diverse regions.

The purpose of this study was to examine the temporal relationship between El Niño and the occurrence of dengue epidemics, and to construct Poisson autoregressive models for the incidences of dengue cases. We analyzed ENSO records, dengue surveillance data, and local meteorological data in two geographically diverse regions in Thailand -- the tropical southern coastal region and the northern inland mountainous region.

## Methods

### Study areas

**Figure 1:**
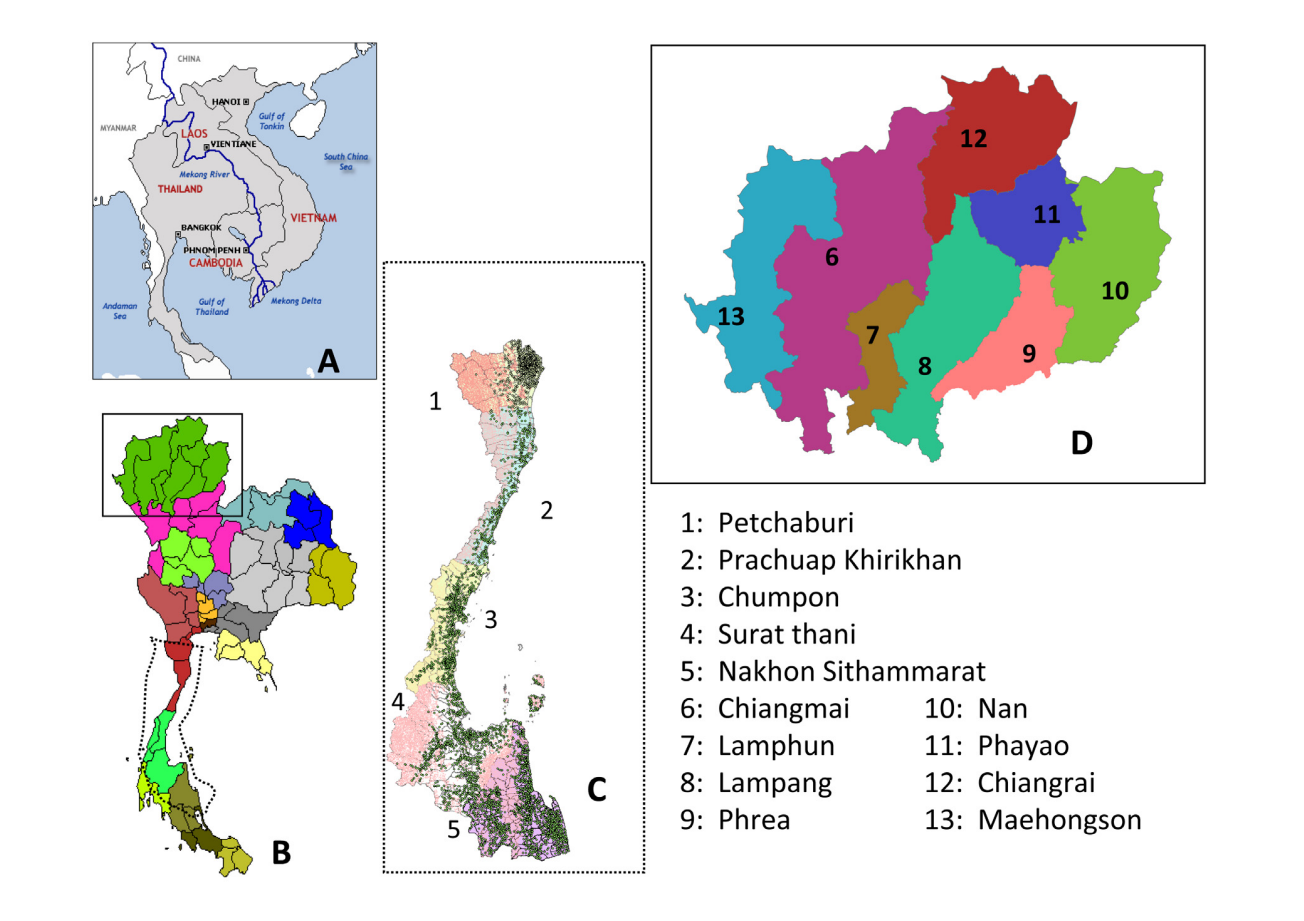
**Study regions**. Study regions and the Gulf of Thailand (A and B). The five provinces (C) in the coastal region and the eight provinces (D) in the northern inland region are numbered. Townships in the coastal region are shown as green spots.

The southern coastal region includes five provinces facing the Gulf of Thailand (Figure 1) with a total population of 3,741,656 people over an area of 41,436 square kilometers. The climate in this region is tropical, with three seasons: (1) summer, mid-February to mid-May; (2) the rainy season (associated with the southwestern monsoon), mid-May to mid-October; and (3) the cool season, mid-October to mid-February. In the cool season, the northeastern monsoon continues to bring scattered rain and high humidity [24].

The northern inland mountainous region includes eight provinces (Figure 1) with a total population of 5,746,545 people over an area of 85,852 square kilometers. There are also three seasons in this region: (1) summer, March to May; (2) the rainy season, May to October; and (3) the cool season, November to February. There were significant temperature fluctuations between day and night, particularly during the summer. During the cool season, this region is relatively dry in comparison with other parts of Thailand [24].

**Figure 2:**
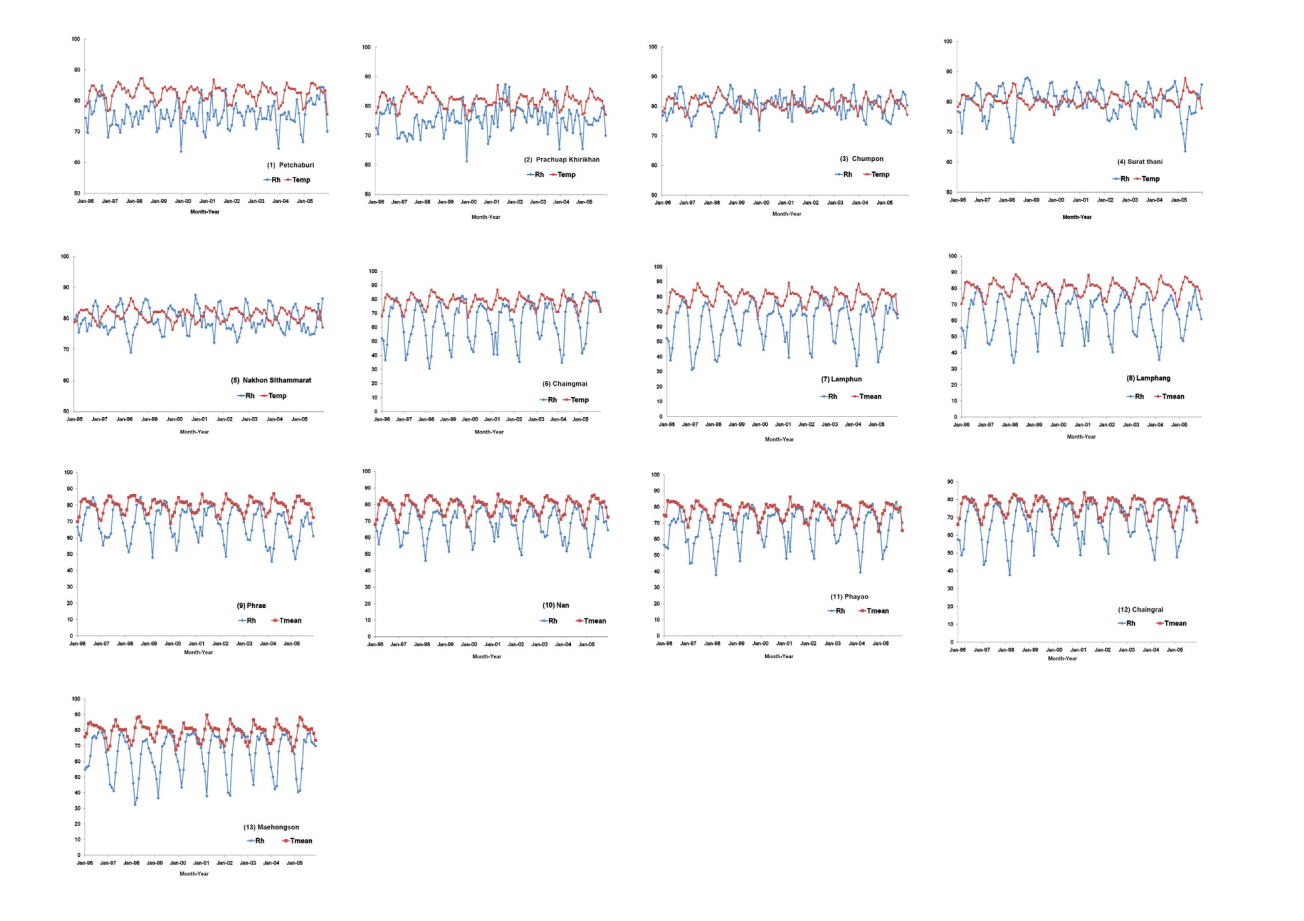
**Local meteorological data, 1996-2005**. Monthly average daily mean temperature (Temp) (red line, units: degrees Fahrenheit) and monthly average daily relative humidity (Rh) (blue line, units: percentage) in (1) Petchaburi; (2) Prachuap Khirikhan; (3) Chumpon; (4) Surat thani; (5) Nakhon Sithammarat; (6) Chaingmail; (7) Lamphun; (8) Lamphang; (9) Phrae; (10) Nan; (11) Phayao; (12) Chaingrai; and (13) Maehongson.

The population data in each province were released by the Ministry of Interior (Bangkok, Thailand) for public use. The mean daily temperature and relative humidity of each province in the above two regions are shown in Figure 2.

### Surveillance for dengue cases

Thailand has had a well-established surveillance system for dengue since 1967. Public hospitals and clinics are required to report all identified dengue cases (based on World Health Organization (WHO) clinical criteria [25-27]) to the Bureau of Epidemiology, Ministry of Public Health (Bangkok, Thailand) on a weekly basis. A case of dengue fever is defined by the presence of acute fever plus at least two of the following clinical findings: high fever, severe headache, back-eye pain, muscle pain, positive tourniquet test, and a white blood cell count of <5,000/μL [27]. A case of dengue hemorrhagic fever is defined by the presence of acute fever with a positive tourniquet test, one of the clinical findings mentioned above, and a 10-20% elevation of hematocrit. Approximately 10-50% of all the reported cases were serologically confirmed [27]. The Bureau of Epidemiology publishes the total numbers of monthly reported dengue cases by each administrative region in the annual surveillance reports [27]. Data since 1996 are accessible from a website and are available for public use.

### Definition of dengue epidemic

**Figure 3:**
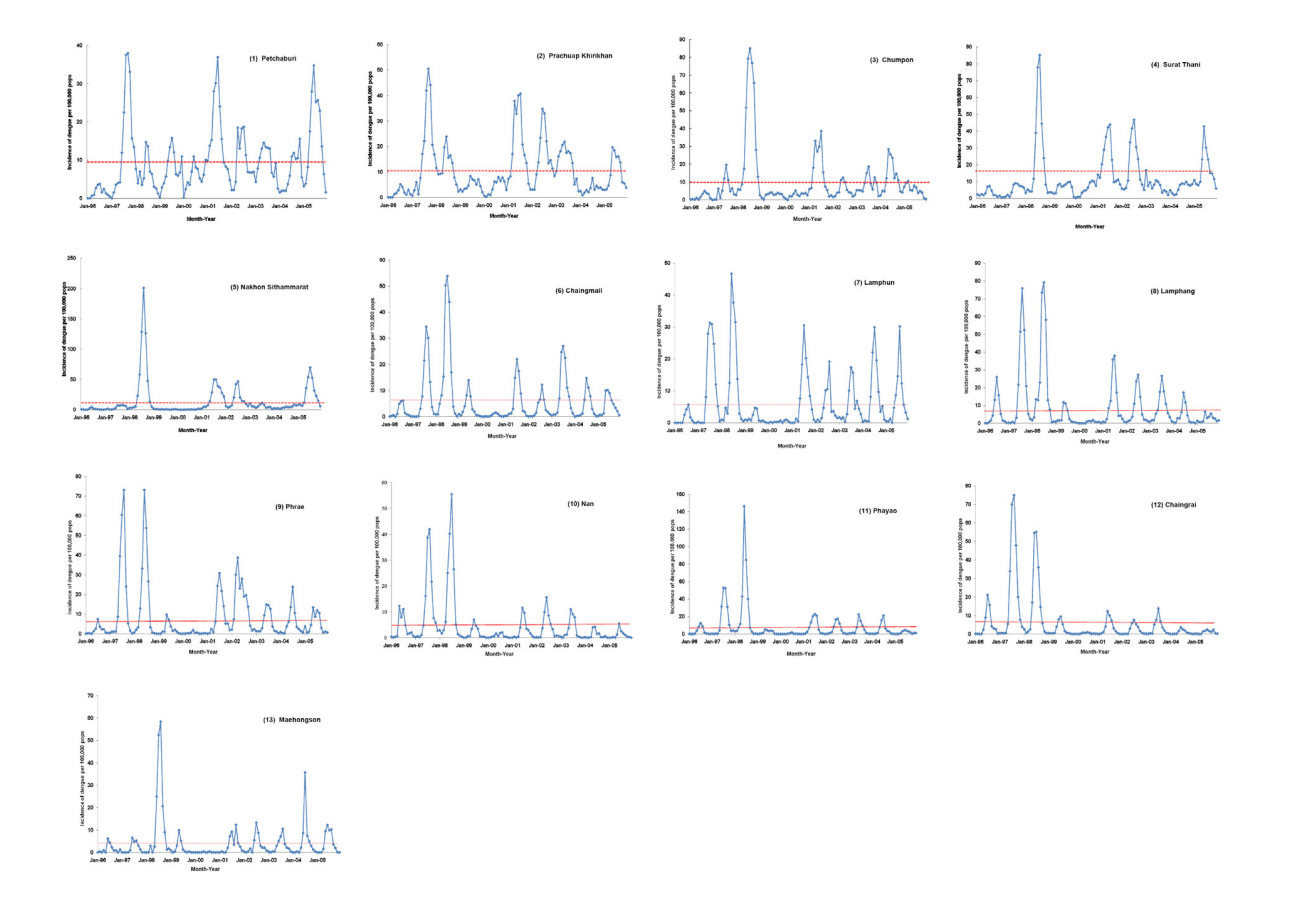
**Dengue surveillance data, 1996-2005**. Monthly incidence of reported dengue cases per 100,000 population in (1) Petchaburi; (2) Prachuap Khirikhan; (3) Chumpon; (4) Surat Thani; (5) Nakhon Sithammarat; (6) Chaingmail; (7) Lamphun; (8) Lamphang; (9) Phrae; (10) Nan; (11) Phayao; (12) Chaingrai; and (13) Maehongson. The red dashed line indicates the 10-year-mean in each province.

The word *epidemic *means "an increase in the number of cases of a disease above what is expected" [28]. Because dengue is a disease endemic to Thailand [27], we define that a province has a dengue epidemic when the monthly incidence of all reported cases, including both dengue fever and dengue hemorrhagic fever, exceeds the provincial 10-year mean from 1996 through 2005. The 10-year mean monthly incidence was calculated by province, as indicated by the red dashed line in Figure 3, rather than aggregated by region.

### El Niño and meteorological data

We obtained data on two ENSO indicators -- the multivariate ENSO index (MEI) and the sea level pressure index (SLP) -- from public databases of the National Oceanic and Atmospheric Administration (Washington, D.C., United States) [29]. The MEI is a comprehensive index calculated from six measurements: sea level pressure, zonal and meridional wind components, sea surface and air temperatures, and total cloudiness, while the SLP is the anomalies of sea level pressure between western and eastern Pacific. The numerical values of both indices are normalized (in the opposite direction) such that an MEI > 1 (or an SLP < -1) defines an occurrence of El Niño [29]. A more positive value of the MEI (or a more negative value of the SLP) indicates a stronger El Niño.

We obtained local climate data from the Department of Meteorology (Bangkok, Thailand). In each province of the study areas, meteorological stations recorded daily weather data, including maximum, minimum and mean temperatures, relative humidity, and wind speed (Table 1). The data on precipitation were incomplete, however, and were not included in the present study. For each province, we used weather data from the station located in its metropolitan area to analyze the impact of local climate on the occurrence of dengue.

**Table 1:** Variable abbreviations

Abbreviation	Definition (Unit)
MEI	Multivariate El Nino-Southern Oscillation (ENSO) index
SLP	Anomalous sea level pressure index (mb)
Tmean	Monthly average mean daily temperature (°F)
Tmin	Monthly average minimum daily temperature (°F)
Tmax	Monthly average maximum daily temperature (°F)
Rh	Monthly average daily relative humidity (%)
WDSP	Monthly average mean daily wind speed (knots)
Case	Monthly count of reported dengue cases (cases)
Pop	Population (persons)
Pop density	Population density (persons per square kilometer)
Epidemic	Binary variable: 1 if the number of monthly dengue cases per 100,000 population > 10-year mean of the province; otherwise, the value is 0.
Lag1-12	Prior months, from 1 to 12
sin12	Oscillation function sin (2πt/T), T (period) = 12 months
cos12	Oscillation function cos (2πt/T), T (period) = 12 months

### Statistical methods

Logistic regression was used to analyze the temporal correlation between ENSO indicators and the occurrences of epidemics, adjusting for the effects of seasonality, population density, and provinces. The oscillatory *sine *and *cosine *functions were used to model seasonal variations of dengue cases [30]. Poisson regression was used to analyze the temporal correlation between ENSO indicators and the incidences of dengue cases. Two statistical methods - (1) quasi-likelihood estimation [31] with the variance function of *μ*^2^, where *μ *is the mean of the outcome variable; and (2) regression with the negative binomial model [32] - were used for the overdispersed data. The effect of the ENSO cycle on the incidence of dengue cases was quantified by R^2 ^calculated as (null deviance - residual deviance) divided by the null deviance.

To construct Poisson autoregressive models for incidences of dengue cases in each province, we included the incidence of dengue cases of 1 month lag, seasonality parameters, and the consistently significant climate variables within time lags ≤ 6 months in the maximum model. Backward elimination procedure was used to select a minimally adequate model. The regression model fitted from 1996-2004 data was then used to forecast the incidence of dengue cases in the year 2005. The robustness of the modeling approach was validated by testing the ability of the regression models to predict out-of-fit data.

The details of the regression models used in each analysis are described in the appendix, and the abbreviations of variables are listed in Table 1. The statistical analysis was performed using S-PLUS 8.04 (TIBCO Software Inc., Palo Alto, CA). Two-tailed p < 0.05 was considered statistically significant.

## Results

### Descriptive epidemiology

From January 1996 through December 2005, a total of 54,051 and 44,176 dengue cases were reported in the southern coastal and the northern inland regions, respectively. The monthly cases per 100,000 population in each province are shown in Figure 3, Panel (1)-(13). There were both seasonal and inter-annual fluctuations. The dengue season usually started in April, peaked during June-July, declined in October, and reached the lowest level during December and January in the southern coastal region; while in the northern inland region, dengue season usually started in May and lasted until October, with few cases in the dry cool season between December and February. Epidemics of dengue occurred in the years of 1997-1998, 2001-2002, and 2005. During epidemics, the number of monthly cases could be as high as 10- to 20-fold more than the usual level.

### Influences of ENSO on local climate

**Figure 4:**
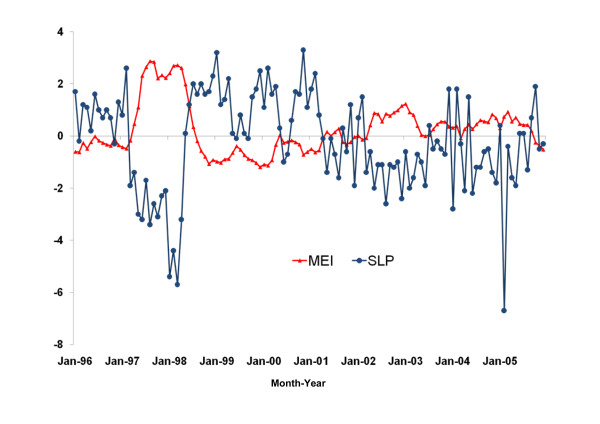
**El Niño Southern Oscillation (ENSO) data, 1996-2005**. The monthly multivariate ENSO index (MEI) (red line with rectangles) and the anomalies of the sea level pressure index (SLP) (blue line with bars). MEI > 1 (or SLP < -1) defines the occurrence of El Niño. A more positive value of MEI (or a more negative value of SLP) indicates a stronger El Niño.

ENSO records from 1996 through 2005 are shown in Figure 4. The influences of El Niño on the local climate in the two study regions are shown in Table 2. In the 5 southern tropical coastal provinces, MEI was positively correlated with local temperatures but negatively correlated with local relative humidity. In the 8 northern inland mountainous provinces, MEI was also positively correlated with local temperatures, but there was no correlation between MEI and local relative humidity.

**Table 2:** Influence of El Nino on local climate

Region/ Time-lag (month)	Tmax	Tmean	Tmin	Rh	WDSP
**In the five southern coastal provinces**					
MEI Lag 0	0.32*	0.34*	0.21*	-0.15*	NS
MEI Lag 1	0.28*	0.30*	0.20*	-0.13*	NS
MEI Lag 2	0.25*	0.27*	0.17*	-0.09*	NS

**In the eight northern mountainous provinces**					
MEI Lag 0	0.15*	0.20*	0.20*	NS	NS
MEI Lag 1	0.12*	0.13*	0.12*	NS	NS
MEI Lag 2	0.09*	0.10*	0.09*	NS	NS

Data shown are Pearson correlation coefficients between the multivariate ENSO index (MEI) and local climate variables. *Statistically significant; NS, not significant.

Abbreviations: Tmax, maximum daily temperature; Tmean, mean daily temperature; Tmin, minimum daily temperature; Rh, relative humidity; WDSP, wind speed.

### Temporal correlation between ENSO indicators and the occurrence of dengue epidemics

After adjusting for the effects of seasonality, population density, and province, both MEI and SLP were consistently predictors (positively for MEI and negatively for SLP) for the occurrence of dengue epidemics throughout the time lags from 1 month through 11 months in both the 5 southern tropical coastal provinces and the 8 northern inland mountainous provinces (Table 3).

**Table 3:** Correlation between ENSO indicators and the occurrence of dengue epidemics over time lags from 1 to 12 months - local climate parameters are also shown for comparison.

Region/Time-lag (month)	ENSO indices		Local climate parameters				
	
	MEI	SLP	Tmax	Tmean	Tmin	Rh	WDSP
**In the five southern coastal provinces**							
Lag 1	0.51*	-0.14*	0.28*	0.46*	0.47*	NS	NS
Lag 2	0.60*	-0.15*	0.27*	0.44*	0.35*	NS	NS
Lag 3	0.66*	-0.24*	0.35*	0.45*	0.19*	-0.05*	NS
Lag 4	0.68*	-0.22*	0.35*	0.43*	0.16*	-0.07*	NS
Lag 5	0.66*	-0.26*	0.30*	0.32*	NS	-0.07*	NS
Lag 6	0.59*	-0.26*	0.26*	0.25*	NS	-0.07*	NS
Lag 7	0.50*	-0.28*	0.16*	0.19*	NS	NS	NS
Lag 8	0.42*	-0.24*	NS	NS	NS	NS	NS
Lag 9	0.39*	-0.20*	NS	NS	NS	NS	NS
Lag 10	0.36*	-0.17*	NS	NS	NS	NS	NS
Lag 11	0.28*	-0.15*	-0.19*	-0.24*	NS	NS	NS
Lag 12	NS	NS	-0.17*	-0.26*	NS	0.07*	NS

**In the eight northern mountainous provinces**							
Lag 1	1.31*	-0.37*	0.19*	0.25*	0.30*	-0.04*	NS
Lag 2	1.29*	-0.38*	0.23*	0.27*	0.20*	-0.07*	NS
Lag 3	1.26*	-0.39*	0.26*	0.25*	0.12*	-0.09*	NS
Lag 4	1.28*	-0.36*	0.15*	0.09*	NS	-0.06*	NS
Lag 5	1.15*	-0.40*	NS	NS	NS	NS	NS
Lag 6	0.91*	-0.36*	NS	NS	NS	NS	NS
Lag 7	0.85*	-0.27*	NS	NS	NS	NS	NS
Lag 8	0.84*	-0.30*	0.09*	NS	NS	NS	NS
Lag 9	0.88*	-0.23*	NS	NS	NS	NS	NS
Lag 10	0.93*	-0.35*	NS	NS	NS	NS	NS
Lag 11	0.84*	-0.41*	NS	NS	NS	NS	NS
Lag 12	0.60*	-0.37*	NS	NS	NS	NS	NS

**NOTE**. Data shown are the logistic regression coefficient of a lagged variable after adjusting for effects of seasonality, population density, and province.

*Statistically significant; NS, not significant.

Abbreviations: MEI, Multivariate ENSO index (monthly average); SLP, sea level pressure index (monthly average); Tmax, maximum daily temperature (monthly average); Tmin, minimum daily temperature (monthly average); Tmean, mean daily temperature (monthly average); Rh, relative humidity (monthly average); WDSP, wind speed (monthly average).

For comparison, local temperatures (maximal, mean, or minimal daily temperature) were consistently predictors for the occurrence of dengue epidemics within time lags from 1 month through 3 months in both regions (Table 3). There also existed a consistent negative correlation between the local relative humidity and dengue epidemics over the time lag from 3 months through 4 months. In contrast, there was no correlation between local wind speed and dengue epidemics.

### Temporal correlation between ENSO indicators and the incidence of dengue cases

After adjusting for effects of seasonality, population density, and province, both MEI and SLP were consistently predictors (positively for MEI and negatively for SLP) for incidences of dengue cases throughout the time lag from 1 month through 11 months in both the 5 southern tropical coastal provinces and the 8 northern inland mountainous provinces (Table 4).

**Table 4:** Correlation between ENSO indicators and the monthly incidence of dengue cases over time lags from 1 to 12 months - local climate parameters are also shown for comparison.

Region/Time-lag (months)	ENSO indices		Local climate parameters				
	
	MEI	SLP	Tmax	Tmean	Tmin	Rh	WDSP
**In the five southern coastal provinces**							
Lag 1	0.29*	-0.05*	0.14*	0.20*	0.13*	NS	NS
Lag 2	0.36*	-0.07*	0.17*	0.21*	0.11*	NS	NS
Lag 3	0.41*	-0.10*	0.19*	0.22*	0.09*	-0.05*	NS
Lag 4	0.42*	-0.13*	0.18*	0.21*	0.07*	-0.07*	NS
Lag 5	0.41*	-0.15*	0.15*	0.18*	0.06*	-0.06*	NS
Lag 6	0.38*	-0.17*	0.11*	0.16*	0.08*	-0.04*	NS
Lag 7	0.34*	-0.16*	0.07*	0.13*	0.09*	NS	NS
Lag 8	0.29*	-0.13*	NS	0.07*	0.07*	NS	NS
Lag 9	0.26*	-0.11*	NS	NS	NS	NS	NS
Lag 10	0.22*	-0.09*	NS	NS	NS	NS	NS
Lag 11	0.18*	-0.06*	-0.05*	-0.07*	NS	NS	NS
Lag 12	0.13*	NS	-0.06*	-0.10*	-0.06*	0.03*	NS

**In the eight northern mountainous provinces**							
Lag 1	0.58*	-0.19*	0.09*	0.09*	0.08*	NS	NS
Lag 2	0.61*	-0.22*	0.09*	0.11*	0.09*	-0.02*	NS
Lag 3	0.61*	-0.25*	0.10*	0.09*	0.06*	-0.04*	NS
Lag 4	0.57*	-0.23*	0.07*	0.04*	NS	-0.04*	NS
Lag 5	0.55*	-0.22*	0.04*	NS	-0.03*	-0.03*	-0.09*
Lag 6	0.53*	-0.21*	NS	NS	-0.04*	-0.03*	-0.13*
Lag 7	0.52*	-0.20*	0.05*	NS	NS	-0.04*	-0.13*
Lag 8	0.49*	-0.18*	0.04*	NS	NS	-0.03*	NS
Lag 9	0.46*	-0.18*	NS	NS	NS	-0.02*	NS
Lag 10	0.40*	-0.16*	0.01*	NS	NS	NS	NS
Lag 11	0.33*	-0.12*	NS	NS	NS	NS	NS
Lag 12	0.25*	-0.17*	NS	NS	NS	-0.40*	0.09*

**NOTE**. Data shown are the Poisson regression coefficients (using quasi-likelihood estimation with the variance function = *μ*^2) of a lagged variable after adjusting for effects of seasonality, population density, and province.

*Statistically significant; NS, not significant.

Abbreviation: MEI, Multivariate ENSO index (monthly average); SLP, sea level pressure index (monthly average); Tmax, maximum daily temperature (monthly average); Tmin, minimum daily temperature (monthly average); Tmean, mean daily temperature (monthly average); Rh, relative humidity (monthly average); WDSP, wind speed (monthly average).

For comparison, local temperatures (maximal, mean, or minimal daily temperature) were also consistently predictors for the incidence of dengue cases within time lags from 1 month through 3 months in both regions (Table 4). There also existed a consistent negative correlation between the local relative humidity and the incidence of dengue cases within time lags from 3 months through 6 months. In contrast, there was no consistent correlation between the monthly average local wind speed and the incidence of dengue cases.

Analyses using the quasi-likelihood estimation (Table 4) and those using negative binomial models (Table 5) yielded the same patterns of temporal correlation between climate variables and incidences of dengue cases with only minor exceptions. The Pearson's correlation coefficient between the regression coefficients obtained from analysis using quasi-likelihood estimation and that from analysis using negative binomial models were 0.99 for MEI lag 1-11 months, and 0.98 for SLP lag 1-11 months, respectively.

**Table 5:** Correlation between ENSO indicators and the monthly incidence of dengue cases over time lags from 1 to 12 months - local climate parameters are also shown for comparison.

Region/Time-lag (month)	ENSO indices		Local climate parameters				
	
	MEI	SLP	Tmax	Tmean	Tmin	Rh	WDSP
**In the five southern coastal provinces**							
Lag 1	0.29*	-0.05*	0.14*	0.20*	0.14*	NS	NS
Lag 2	0.36*	-0.07*	0.17*	0.21*	0.11*	NS	NS
Lag 3	0.40*	-0.10*	0.19*	0.22*	0.09*	-0.04*	NS
Lag 4	0.42*	-0.12*	0.18*	0.21*	0.07*	-0.05*	NS
Lag 5	0.41*	-0.15*	0.15*	0.18*	0.06*	-0.05*	NS
Lag 6	0.38*	-0.17*	0.11*	0.16*	0.08*	-0.03*	NS
Lag 7	0.34*	-0.16*	0.07*	0.13*	0.09*	NS	NS
Lag 8	0.29*	-0.12*	NS	0.08*	0.07*	NS	NS
Lag 9	0.26*	-0.11*	NS	NS	NS	0.02*	NS
Lag 10	0.22*	-0.09*	NS	NS	NS	0.02*	NS
Lag 11	0.19*	-0.7*	-0.05*	-0.07*	NS	0.02*	NS
Lag 12	0.13*	NS	-0.06*	-0.10*	-0.06*	0.02*	NS

**In the eight northern mountainous provinces**							
Lag 1	0.58*	-0.18*	0.08*	0.09*	0.07*	-0.01*	-0.08*
Lag 2	0.62*	-0.22*	0.09*	0.10*	0.09*	-0.02*	NS
Lag 3	0.61*	-0.25*	0.10*	0.10*	0.06*	-0.04*	NS
Lag 4	0.57*	-0.23*	0.07*	0.05*	NS	-0.04*	NS
Lag 5	0.54*	-0.23*	0.05*	0.01*	-0.04*	-0.03*	NS
Lag 6	0.51*	-0.21*	0.03*	NS	-0.05*	-0.03*	NS
Lag 7	0.48*	-0.19*	0.04*	NS	-0.03*	-0.04*	NS
Lag 8	0.46*	-0.17*	0.03*	NS	NS	-0.03*	NS
Lag 9	0.43*	-0.17*	NS	NS	NS	-0.02*	NS
Lag 10	0.38*	-0.16*	NS	NS	NS	NS	NS
Lag 11	0.32*	-0.13*	NS	NS	NS	NS	NS
Lag 12	0.24*	-0.11*	NS	NS	NS	NS	0.11*

**NOTE**. Data shown are the Poisson regression coefficients (using the negative binomial model) of a lagged variable after adjusting for effects of seasonality, population density, and province.

*Statistically significant; NS, not significant.

Abbreviation: MEI, Multivariate ENSO index (monthly average); SLP, sea level pressure index (monthly average); Tmax, maximum daily temperature (monthly average); Tmin, minimum daily temperature (monthly average); Tmean, mean daily temperature (monthly average); Rh, relative humidity (monthly average); WDSP, wind speed (monthly average).

### Effects of ENSO cycle on monthly incidence of dengue cases

Effects of ENSO alone, local climate alone, or both over the past 6 months on the incidence of dengue cases are shown in Table 6. Up to 22% (in 8 northern inland mountainous provinces) and 15% (in 5 southern tropical coastal provinces) of the variation in the monthly incidence of dengue cases was attributable to the global ENSO cycles alone.

**Table 6:** Effects of global ENSO cycles on the monthly incidence of dengue cases

Model*	R^2^
Southern tropical coastal region	
Global index model	0.15
Local climate model	0.29
Combined model	0.33
North inland mountainous region	
Global index model	0.22
Local climate model	0.57
Combined model	0.69

*see Appendix for details. Global index model, model with multivariate ENSO index (MEI) lagged 1-6 months; Local climate model, model with local temperature lagged 1-3 months and relative humidity lagged 3-6 months; Combined model, model with both MEI and local temperature/relative humidity.

### Validation of models using out-of-fit data in each province

**Figure 5:**
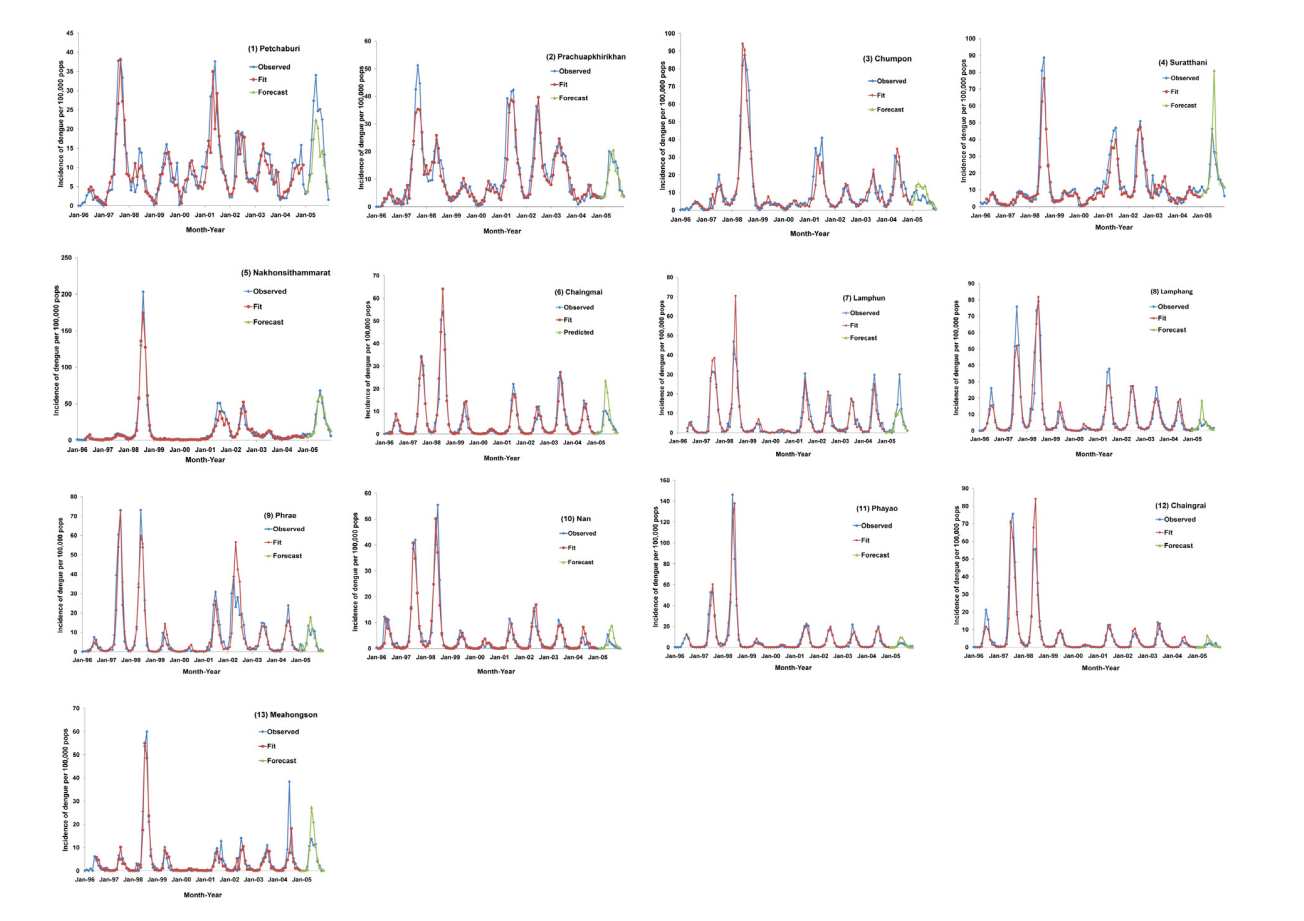
**Observed (1996-2005), fitted (1996-2004), and forecasted (2005) monthly incidence of reported dengue cases**. Observed (blue line, 1996-2005), fitted (red line, 1996-2004), and forecasted (green line, 2005) monthly incidence of reported dengue cases. (1) Petchaburi; (2) Prachuap Khirikhan; (3) Chumpon; (4) Surat thani; (5) Nakhon Sithammarat; (6) Chaingmail; (7) Lamphun; (8) Lamphang; (9) Phrae; (10) Nan; (11) Phayao; (12) Chaingrai; (13) Maehongson.

We fitted regression models for incidences of dengue cases using 1996-2004 data from each province and evaluated the ability of fitted models to forecast out-of-fit data in the year 2005. The details of the models varied across different provinces, but MEI remained an independent predictor in 10 out of the 13 studied provinces (Table 7). Monthly incidences of dengue cases predicted by the fitted models were well matched to those actually reported in each province during the year 2005 (Figure 5). The percentage of correct predictions by the fitted models for the occurrence or the absence of dengue epidemics in the 12 months during 2005 were 83-100% in 12 out of the 13 studied provinces (Table 8).

**Table 7:** Province-specific models for the incidences of dengue cases, fitted from 1996-2004 data.

Province	Variable	Regression coefficient	Standard error	R^2^
**1. Petchaburi**	Constant	-10.933	1.351	0.72
	ln(Population)	1	(offset)	
	ln(Case Lag1/Pop Lag1)	0.351	0.007	
	Rh Lag3	-0.016	0.007	
	Tmean Lag1	0.051	0.013	
	MEI Lag2	0.088	0.038	
	MEI Lag5	-0.110	0.037	
	sin12	0.098	0.039	

**2. Prachuap Khirikhan**	Constant	-7.553	0.255	0.83
	ln(Population)	1	(offset)	
	ln(Case Lag1/Pop Lag1)	0.393	0.027	
	MEI Lag2	0.226	0.064	
	MEI Lag3	-0.184	0.064	
	cos12	-0.150	0.032	
	sin12	0.069	0.033	

**3. Chumpon**	Constant	-5.915	0.831	0.81
	ln(Population)	1	(offset)	
	ln(Case Lag1/Pop Lag1)	0.324	0.035	
	Rh Lag3	-0.029	0.010	
	MEI Lag6	0.088	0.030	
	cos12	-0.189	0.054	
	sin12	0.094	0.046	

**4. Surat Thani**	Constant	-5.621	0.493	0.84
	ln(Population)	1	(offset)	
	ln(Case Lag1/Pop Lag1)	0.426	0.023	
	Rh Lag3	-0.024	0.006	
	cos12	-0.081	0.038	
	sin12	0.093	0.036	

**5. Nakhon Sithammarat**	Constant	-3.147	0.693	0.93
	ln(Population)	1	(offset)	
	ln(Case Lag1/Pop Lag1)	0.443	0.017	
	Rh Lag3	-0.029	0.008	
	Rh Lag4	-0.028	0.009	
	cos12	-0.191	0.035	
	sin12	0.243	0.049	

**6. Chaingmai**	Constant	-11.158	1.023	0.89
	ln(Population)	1	(offset)	
	ln(Case Lag1/Pop Lag1)	0.326	0.022	
	Tmean Lag2	0.026	0.011	
	MEI Lag1	0.124	0.027	
	cos12	-0.503	0.052	

**7. Lamphun**	Constant	-16.343	2.193	0.79
	ln(Population)	1	(offset)	
	ln(Case Lag1/Pop Lag1)	0.304	0.043	
	Rh Lag5	0.011	0.005	
	Tmean Lag1	0.045	0.017	
	Tmean Lag3	0.042	0.015	
	MEI Lag1	0.127	0.041	
	cos12	-0.364	0.091	

**8. Lamphang**	Constant	-10.794	0.994	0.88
	ln(Population)	1	(offset)	
	ln(Case Lag1/Pop Lag1)	0.340	0.032	
	Tmean Lag2	0.029	0.011	
	MEI Lag1	0.118	0.030	
	cos12	-0.395	0.066	
	sin12	0.189	0.071	

**9. Phrae**	Constant	-7.623	0.342	0.84
	ln(Population)	1	(offset)	
	ln(Case Lag1/Pop Lag1)	0.402	0.033	
	MEI Lag2	0.307	0.088	
	MEI Lag3	-0.236	0.086	
	cos12	-0.417	0.067	
	sin12	0.257	0.063	

**10. Nan**	Constant	-14.249	1.712	0.81
	ln(Population)	1	(offset)	
	ln(Case Lag1/Pop Lag1)	0.243	0.033	
	Tmean Lag1	0.059	0.019	
	MEI Lag1	0.208	0.039	
	cos12	-0.472	0.094	

**11. Phayao**	Constant	-8.137	0.538	0.89
	ln(Population)	1	(offset)	
	ln(Case Lag1/Pop Lag1)	0.296	0.041	
	Rh Lag6	-0.012	0.005	
	MEI Lag2	0.199	0.042	
	cos12	-0.762	0.076	
	sin12	0.250	0.106	

**12. Chaingrai**	Constant	-17.189	1.954	0.90
	ln(Population)	1	(offset)	
	ln(Case Lag1/Pop Lag1)	0.359	0.035	
	Tmean Lag1	0.072	0.020	
	Tmean Lag2	0.039	0.019	
	MEI Lag1	0.127	0.035	
	cos12	-0.283	0.089	
	sin12	0.395	0.113	

**13. Maehongson**	Constant	-16.716	1.614	0.67
	ln(Population)	1	(offset)	
	ln(Case Lag1/Pop Lag1)	0.405	0.060	
	Rh Lag5	-0.022	0.008	
	Rh Lag6	0.023	0.008	
	Tmean Lag1	0.115	0.019	

**Table 8:** The percentage of correct predictions by the fitted models for the occurrence or the absence of dengue epidemics in 2005

Province	Ratio of correct prediction*
**1. Petchaburi**	11/12 (92%)
**2. Prachuap Khirikhan**	12/12 (100%)
**3. Chumpon**	8/12 (67%)
**4. Surat Thani**	10/12 (83%)
**5. Nakhon Sithammarat**	12/12 (100%)
**6. Chaingmai**	11/12 (92%)
**7. Lamphun**	10/12 (83%)
**8. Lamphang**	11/12 (92%)
**9. Phrae**	11/12 (92%)
**10. Nan**	10/12 (83%)
**11. Phayao**	10/12 (83%)
**12. Chaingrai**	11/12 (92%)
**13. Maehongson**	11/12 (92%)

*For the occurrence or the absence of dengue epidemics in the 12 months during 2005 in each province.

## Discussion

Our results show a consistent temporal correlation of ENSO indicators with both epidemics of dengue and incidences of dengue cases throughout time lags of 1-11 months over both the southern tropical coastal and northern inland mountainous regions in Thailand from 1996 through 2005. Up to 22% (northern region) and 15% (coastal region) of the variation in the monthly incidence of dengue cases was attributable to global ENSO cycles. Our findings strongly support the hypothesis that El Niño is one of the important driving forces of dengue epidemics across the geographically diverse regions in Thailand [8,9,12,13].

The province-level analysis in the present study also highlights the spatial heterogeneity of the effect of the ENSO on the incidence of dengue cases, as evidenced by the differences in the province-specific models. The ENSO indicator remained an independent predictor in 10 out of the 13, but not in all studied provinces. This observation is in keeping with of a recent investigation in Puerto Rico [15] that revealed that the effect of climate on dengue transmission on a local scale may differ from global expectations.

The feasibility of determining the association between the ENSO and the incidence of dengue cases with simple statistical models has been previously explored with varying success [12,13,15,33]. The overdispersion in counts of dengue cases, which violates the assumptions of many popular statistical models, requires appropriate approaches in model fitting [31,32]. A well-established method is to use the negative binomial model, which has one more parameter than the Poisson model; the second parameter can be used to adjust the variance independently of the mean [32]. Another well-established method is to use quasi-likelihood estimation based on the variance-mean relationship without making parametric assumptions about the error distribution [31], particularly when investigators are not sure whether the distribution is indeed a negative binomial. To verify the robustness of the assessment, we compared results obtained by the two approaches. Both yielded the same patterns of temporal correlation between the climate variables and the incidences of dengue cases with only minor differences that did not affect the conclusions. Furthermore, the logistic regression analysis yielded the same pattern of temporal correlation between the climate variables and the dengue epidemics. The robustness of the modeling method was further verified by the demonstration of the predictive ability of the regression model fitted from 1996-2004 data for out-of-fit 2005 data.

In addition to supporting a causal effect of El Niño on dengue epidemics, our data indicate that the ENSO and local climate together explain 33-69% of the variation in the incidence of dengue cases in the studied regions (Table 6). The remaining 31-67% of the variation possibly involved non-climatic causes, such as population immunity [16-19] and socio-environmental factors that influence the breeding and ecology of mosquito vectors [20-23]. Nevertheless, the incorporation of both El Niño and local climate data into province-specific models (using the incidence of dengue cases in the previous month as the baseline) does achieve an R^2 ^in the range of 0.67-0.93 (Table 7), a good match between the out-of-fit forecasted incidences of dengue cases and those actually reported in the year 2005, and an 83-100% correct predictions for the occurrence or the absence of dengue epidemics in 12 of the 13 provinces during 2005 (Figure 5 and Table 8).

The effect of the ENSO on dengue epidemics is probably mediated primarily through its warming effect on local temperature, which in turn enhances the replication of the dengue virus and the biting behavior of the mosquito vector *Aedes aegypti *[10,11]. There is a positive association between mosquito indices and increases in local temperature in the previous month [34,35]. Therefore, our findings should not be extrapolated to regions where *Aedes aegypti *is not present [21]. Although global warming may contribute to the geographic expansion of dengue-affected areas [36,37], climatic factors alone are unlikely to cause dengue epidemics in the absence of vectors [22].

Besides the warming effect, El Niño has caused extensive drought in western Pacific regions [5-7]. In the coastal region, MEI was negatively correlated with local relative humidity (Table 2). The effect of drought on dengue transmission is more complex than that mediated through temperature. Rainfall increases the range of the natural habitat suitable for mosquito breeding and can facilitate dengue transmission [8,9,20,38]; however, mosquitoes can still breed in sites where there is water storage during droughts [1,21]. In northeast Thailand, the 1987 dengue hemorrhagic fever epidemic occurred during a dry, hot season but stopped before the arrival of rainy season [20]. Relative humidity is an indicator of the likelihood of precipitation. The present study also shows that the monthly average local relative humidity in the prior 3-6 months was negatively associated with epidemics of dengue and incidences of dengue cases in both the southern coastal and northern inland mountainous regions of Thailand.

In addition to the induction time from the increase in temperature/water storage to the shortening of the extrinsic incubation period of the virus in the mosquito and the acceleration of the production of adult mosquitoes, there were additional time lags from the increase in dengue transmission to the outbreak of dengue epidemics. There were also time lags from the onset of clinical illness to the reporting to the Ministry of Health. On the other hand, we did acknowledge that the climate conditions 7-12 months ago might be less likely to affect current dengue transmission, and therefore excluded these from the ENSO effect estimation model and province-specific predictive models.

There were two limitations in the present study. First, because we did not have serological and entomological data, we were unable to empirically determine the induction time between El Niño and the onset of the increase in dengue transmission. Second, the dengue surveillance data used in the present study were based on reports from hospitals and clinics, and they may be an underestimation of the true incidence of dengue infection in the study regions. Furthermore, the clinical criteria used to define dengue cases did not have perfect sensitivity and specificity. Nevertheless, the Thailand dengue surveillance system consistently used case definitions based on well-established WHO criteria and employed a stable and systemic data collection process, making the numbers of dengue cases from different months and provinces directly comparable during the study period [25-27].

Because Thailand is a dengue endemic area and has a tropical climate strongly influenced by oceanographic phenomena, the effects of El Niño on dengue epidemics observed in our study may not be generalizable to non-tropical countries where dengue is not endemic and the ecological factors for mosquito vectors are different. Future investigations on modeling dengue surveillance data elsewhere may provide more information on the complex interactions between El Niño and infectious diseases.

## Conclusion

El Niño is one of the important driving forces of dengue epidemics across geographically diverse regions in Thailand; however, spatial heterogeneity in the effect is present. The effects of El Niño should be taken into account in future epidemic forecasting for public health preparedness.

## Competing interests

The authors declare that they have no competing interests.

## Authors' contributions

MT acquired the publically available dengue surveillance data, El Niño and local climate data in Thailand, analyzed data, interpreted the findings, and drafted the manuscript. CTF analyzed data, interpreted the findings, and revised the manuscript. SK acquired the publically available dengue surveillance data and local climate data in Thailand. CML initiated the research grant proposal and obtained the funding. CCK co-initiated the research grant proposal and revised the manuscript. All authors read and approved the final manuscript.

## Appendix

1. Modeling the correlation of a lagged climate variable with dengue epidemics

The models were:

Epidemic ~ sin12 + cos12 + ∑ dummy variables for provinces + population density + tested lagged climate variable, link = logistic, family = binomial

2. Modeling the correlation of a lagged variable with incidences of dengue cases

The models were:

Case ~ offset ln(population) + sin12 + cos12 + ∑ dummy variables for provinces + population density + tested lagged variable, link = log, family = quasi(variance = *μ*^2) or family = negative binomial

3. Modeling the incidence of dengue cases in each province

The initial maximum model for each province was:

Case^1/2^~offset ln(population) + ln(Case Lag1/Pop Lag1) + sin12 + cos12 + ∑ MEI Lag(1-6) + ∑ Tmean Lag(1-3) + ∑ Rh Lag(3-6), link = log, family = quasi (variance = *μ*)

The square root transformation of the dependent variable allowed the use of a variance function of *μ*. The backward elimination procedure was used to select a minimally adequate model for each province. The final models retained only the covariates with statistically significant regression coefficients and the ln(population) as the offset.

4. Modeling the effect of climate on the incidence of dengue cases

The models were:

Global index model: Case ~ offset ln(population) + ∑ MEI Lag (1-6), link = log, family = quasi(variance = *μ*^2)

Local climate model: Case ~ offset ln(population) + ∑ Tmean Lag(1-3) + ∑ Rh Lag(3-6), link = log, family = quasi(variance = *μ*^2)

Combined model: Case ~ offset ln(population) + ∑ MEI Lag (1-6) + ∑ Tmean Lag(1-3) + ∑ Rh Lag(3-6), link = log, family = quasi(variance = *μ*^2)

## Pre-publication history

The pre-publication history for this paper can be accessed here:

http://www.biomedcentral.com/1471-2458/9/422/prepub
